# A mouse model study of toxicity and biodistribution of a replication defective adenovirus serotype 5 virus with its genome engineered to contain a decoy hyper binding site to sequester and suppress oncogenic HMGA1 as a new cancer treatment therapy

**DOI:** 10.1371/journal.pone.0192882

**Published:** 2018-02-20

**Authors:** Faizule Hassan, Sarah L. Lossie, Ellen P. Kasik, Audrey M. Channon, Shuisong Ni, Michael A. Kennedy

**Affiliations:** Department of Chemistry and Biochemistry, Miami University, Oxford, Ohio United States of America; Justus Liebig Universitat Giessen, GERMANY

## Abstract

The HGMA1 architectural transcription factor is highly overexpressed in many human cancers. Because HMGA1 is a hub for regulation of many oncogenes, its overexpression in cancer plays a central role in cancer progression and therefore HMGA1 is gaining increasing attention as a target for development of therapeutic approaches to suppress either its expression or action in cancer cells. We have developed the strategy of introducing decoy *hyper binding sites* for HMGA1 into the nucleus of cancer cells with the goal of competetively sequestering overexpressed HMGA1 and thus suppressing its oncogenic action. Towards achieving this goal, we have introduced an HMGA1 decoy *hyper binding site* composed of six copies of a high affinity HMGA1 binding site into the genome of the replication defective adenovirus serotype 5 genome and shown that the engineered virus effectively reduces the viability of human pancreatic and cancer cells. Here we report the first pre-clinical measures of toxicity and biodistribution of the engineered virus in C57BL/6J Black 6 mice. The immune response to exposure of the engineered virus was determined by assaying the serum levels of key cytokines, IL-6 and TNF-α. Toxicity due to exposure to the virus was determined by measuring the serum levels of the liver enzymes aspartate aminotransferase and alanine aminotransferase. Biodistribution was measured following direct injection into the pancreas or liver by quantifying viral loads in the pancreas, liver, spleen and brain.

## Introduction

Recombinant adenovirus (Ad) vectors are widely used for gene delivery, cancer treatment and as vaccines to express antigenic peptides [1]. More than 400 gene therapy trials have been or are being performed with Ad vectors [1]. The use of adenovirus vectors in cancer treatment has been approved in China since 2004 [2]. While there are at least 57 serotypes of human Ad (Ad1-Ad57), Ad5 is the most common serotype used in gene delivery [1]. Ad5 is a non-enveloped virus which has a double stranded DNA genome and an icosahedral capsid of primarily penton and hexon proteins [3]. The ability to accommodate long foreign DNA sequences and to infect both dividing and non-dividing cells of different types efficiently make adenovirus vectors excellent tools for gene therapy and cancer treatment [4]. In addition, Adenovirus can be prepared as a high titer viral stock, and the dsDNA genome is not integrated into the host cell chromosome [1].

Ad vectors used for gene therapy consist of two categories, replication competent and replication defective [5]. Replication defective vectors are useful in clinical treatment because of their reduced immune response and toxicity [6]. Deletion of the E1 and E3 regions of Ad vector renders the virus unable to replicate and also allows the virus to carry foreign DNA up to 7.5 kb in size [3]. The E1 region contains information crucial to virus replication and the products from the E3 region have been shown to elicit host immune system [1]. Deletions in these regions result in a safer, more effective vector that is capable of delivering foreign DNA to cells *in vivo*.

One of the major limitations associated with the clinical use of Ad vectors is that they can elicit a strong immunogenic response *in vivo [7].* The immunogenic response against Ad involves both innate and adaptive immune responses [8]. The innate immune response to Ad is initiated through the release of cytokines almost immediately after infection and is the major barrier to Ad clinical treatment [4, 7]. Viral interaction with Kupffer cells, macrophages, and dendritic cells results in the release of cytokines such as IL-6, TNFα, and IP-10, and RANTES [8]. Additional inflammatory cytokines including IFNγ, IL-1β, and IL-12 and a number of chemokines (such as MIP-1α and MIP-2) have also been found to contribute to the adenovirus mediated immune response in humans [1]. Among the cytokines, IL-6 and TNFα are two of the major contributors of Ad induced inflammatory response and liver toxicity [8, 9]. Blocking of IL-6 receptor or TNFα receptor before Ad administration has been shown to significantly reduce liver toxicity in mice [8, 10]. Liver enzymes such as aspartate transaminase (AST) and alanine transaminase (ALT) which are used as biochemical indicators of liver damage, were found to be increased in numerous studies as a consequence of Ad induced liver damage [11]. Ad modifications such as PEGylation with polyethylene glycol (PEG) or fiber modifications have been shown to decrease the innate immune response to the virus and liver inflammation [8].

High Mobility Group A (HMGA) is an *architectural transcription factor* that binds to double-stranded DNA using a DNA binding domain known as “AT hooks”. HMGA binds to the minor groove of DNA in AT rich regions [12–15], promoting conformational changes in the DNA structure in the context of chromatin [16], and therefore it is also known as a chromatin remodeling factor. The AT rich HMGA binding regions consist of multiple consecutive adenine or thymine nucleotides [17, 18]. Upon binding to these regions, HMGA recruits other protein molecules and play an important role in numerous cellular pathways including regulation of gene expression, DNA repair, cell proliferation, apoptosis, etc. [19, 20]. HMGA plays an essential role in embryonic development and is therefore expressed at high levels in embryonic tissues [21]. The expression of HMGA has been shown to be very low or undetectable in normal adult tissues [22]. However, overexpression HMGA has been reported in almost every type of human cancer [23]. Overexpression of *HMGA* is known to stimulate cancer cell proliferation and contribute to tumor growth [23] by suppressing apoptotic function of the tumor suppressor protein, p53 [24]. In addition, HMGA has been found to induce chemotherapy resistance in pancreatic cancer [25]. We have previously shown that transfection of human pancreatic cancer cells with phosphorothioate substituted DNA aptamers containing single 15-base pair HMGA decoy binding sites reduced cancer cell viability after chemotherapy treatment [26].

We have designed a non-naturally-occurring decoy *hyper binding site* for HMGA, described in our previous study [27], consisting of six copies of an individual HMGA binding site, which we refer to as HMGA-6. We have engineered a replication defective adenovirus vector (referred as AdEasy-HMGA-6) to carry and deliver the decoy HMGA-6 *hyper binding site* to the cancer cell nucleus in order to sequester overexpressed HMGA proteins. The HMGA-6 *hyper binding site* was intended to be used as a decoy for binding excess HMGA in cancer cells nucleus, thereby disrupting the oncogenic consequences caused by HMGA-associated misregulation of gene expression and resistance to chemotherapy. In our earlier study, we have showed that infection of pancreatic and liver cancer cell lines with the AdEasy-HMGA-6 virus significantly reduced cancer cell viability and increased sensitivity to chemotherapy drug gemcitabine [27].

Here, we investigated the toxicity and biodistribution following injection of the AdEasy-HMGA-6 virus into C57BL/6J mice liver or pancreas. Previous research has indicated that upon intravenous administration, Ad is swept to the liver rapidly by binding to platelets and is degraded by Kupffer cells [28]. Although intravenous (IV) injection is often preferred to treat metastases and advanced cancers that persist in multiple tissue types, rapid clearance to the liver upon IV injection results in decreased transduction into the tissues of interest. Cytokine release and pathological damage in animal models has been shown to be heightened upon IV injection of Ad vectors [28]. Injecting the virus intramuscularly or directly into the organ of interest has been suggested to increase viral efficacy by decreasing systemic distribution and the potentially toxic immune response [28]. Therefore, in the current study, the virus was injected directly into the pancreas or liver of mice in order to increase virus transfection in the target organs and decrease the rapid immune response that can diminish viral distribution and increase toxicity *in vivo*. The goal of the current study was to evaluate the *in vivo* toxicity and immune response of our engineered AdEasy-HMGA-6 virus. To achieve these goals, we measured serum levels of key cytokines, IL-6 and TNF-α, as well as liver enzymes aspartate aminotransferase (AST) and alanine aminotransferase (ALT) following virus administration in mice in order to determine if the inclusion of the decoy HMGA-6 *hyper binding site* in a replication defective Ad vector caused any additional toxic effects compared to the empty Ad vector itself. This work provides essential preclinical information regarding toxicity and biodistribution needed to determine the potential of our engineered virus for future treatment of cancer in humans.

## Materials and methods

### Ad vectors

The two Ad vectors (AdEasy and AdEasy-HMGA-6) used in this study were constructed in our lab previously [27]. Briefly, the replication defective Ad containing plasmid (pAdEasy) was purchased from Agilent Technologies and the 148 bp synthetic HMGA-6 *hyper-binding site* was cloned in the pAdEasy vector (called pAdEasy-HMGA-6). The linearized pAdEasy or pAdEasy-HMGA-6 DNA was transfected into the AD293 cell line, a derivative of human embryonic kidney cell line, (Agilent Technologies) which supports the replication of the replication defective Ad to produce infectious virus particles. The viruses were quantified by plaque assay and 1.0X10^8^ plaque forming unit (pfu)/mL concentration stock was used for this study.

### Animal and sample collection

C57BL/6 mice were purchased from the Jackson Laboratory, USA. 6–8 weeks old female mice were used in this study. All procedures were approved by the Miami University Institutional Animal Care and Use Committee and complied with the ARVO statement for use of animals in research and consistent with those published by the Institute for Laboratory Animal Research (Guide for the Care and Use of Laboratory Animals). Mice were anasthesized with 3% isoflurane and subcutaneously injected with 0.05–0.10 mg/kg buprenorphine for pain relief. A 1 cm incision was made in the ventral upper left quadrant of the mouse. The pancreas or liver was exposed at a site near the stomach. 1.0X10^8^ vp/Kg of body weight of AdEasy, AdEasy-HMGA-6 or 20 μL PBS was directly injected into liver or pancreas. This corresponded to about 2 x 10^6^ vp injected into the organ of each mouse for an average mouse weight of 20 g. The dose of 1.0X10^8^ vp/Kg is within the range reported for other toxicity studies involving adenovirus [28, 29]. The idea of dose has a different meaning in this study compared to many other published studies in that the viruses were not administered by systemic delivery by intravenous injection or intramuscular injection, but rather the viruses were administered by direct injection into the target organ to mimic virus injection directly into an organ-specific tumor. Therefore, even though a lower vp/Kg dose was used in this study compared many other reported studies, the dose to the target organ may be comparable or even higher in the study reported here. Because the goal of this study was to compare the toxicity and immunogenicity of the engineered virus to the native virus, the fact that we were able to detect the virus in the target organs, and to detect elevated serum toxicity and immune response markers, validates the dose used in this comparison.

For either organ, mice were divided into four groups: (i) AdEasy, (ii) AdEasy-HMGA-6, (iii) PBS, (iv) Sham control. The mouse body weight was monitored for changes after surgery and compared to its control weight every 12 h for the first 24 h post-surgery (except the 6 h time point group). After the first 48 hours, the mouse's weight was monitored weekly and compared to its control weight. The mice injected in the pancreas or liver was monitored for swelling in the abdominal region and monitored for other behavioral changes that indicate stress or pain, such as head pressing, reduced social interaction, porphyrin secretions, hunched stature, etc. Three mice were euthanized by cardiac puncture at 6 h, 3, 7, 30 days post-injection from each group. Blood sample was collected for serum isolation and tissues were snap frozen in liquid nitrogen immediately and both serum and tissue samples were stored at -80°C until be used for DNA isolation.

### DNA isolation and qRT-PCR

Total DNA was isolated from finely minced tissues (∼20 mg of liver, pancreas, brain and ∼15 mg of spleen) by using DNeasy 96 Blood & Tissue Kit from Qiagen, USA following the manufacturer’s guideline. Quantitive real time PCR was performed to determine the copy number of adenoviral genomic DNA in liver, pancreas, brain and spleen by using iTaq™ Universal SYBR^®^ Green Supermix from Bio-Rad, USA. The following primers were used based on a previous study [30]. The sequences of the forward and reverse primers were 5’-CCACCGATAGCAGTACCCTT-3’ and 5’-GACCAGTTGCTACGGTCAAA-3’, respectively. The PCR cycle consists of one cycle of 95°C for 3 min and then 40 cycles of 95°C for 10 s, 55°C for 10 s and 72°C for 30 s and one cycle of 95°C for 10 s. Purified pAdEasy vector (Agilent Technologies) with known copy number (2.2X10^6^ to 2.2X10^-1^ copy) was used to prepare the standard curve for the determination of Ad viral DNA copy number in the samples. Each sample and standard were run in triplicate. A Bio-Rad CFX connect PCR detection system was used for real time PCR and data was anlyzed by Bio-Rad CFX manager. A negative control using molecular biology grade water (ThermoFisher Scientific, USA) instead of DNA template was included and the quantification cycle (Cq) values less than the negative control were considered as negative.

### Serum cytokines and transaminase level determination

The concentrations of TNFα and IL-6 were determined using the TNF alpha Mouse ELISA Kit and IL-6 Mouse ELISA Kit respectively from ThermoFisher Scientific, USA as per manufacturer’s recommendations. Serum AST and ALT levels were measured using Amplite™ Colorimetric Aspartate Aminotransferase (AST) Assay Kit and Amplite™ Colorimetric Alanine Aminotransferase Assay Kit respectively from AAT Bioquest, USA according to the manufacture’s protocol. For both cytokine and transaminase level determination the absorbance was measured using BioTek Synergy H1 plate reader Instrument. For both assays, each sample and standard were run in duplicate.

### Statistical analysis

The two-tailed unpaired Welch’s T-test was performed to determine the significance between groups. The difference were considered significant when *p* values were less than 0.05. All data on the figures represent the means ± SD of three mice.

## Results

In a previous study, we engineered a decoy HMGA-6 *hyper binding site* into the replication defective adenovirus serotype 5 genome (referred to as AdEasy-HMGA-6) and demonstrated that infection with the engineered virus significantly reduced the viability of four different human pancreatic cancer cells lines and one human liver cancer cell line [27]. In this study, we sought to evaluate the intratumoral injection method (as a model we injected the virus directly into the liver or pancreas) as a potential route of administration of the engineered virus in mice. Specifically, we used this model system to evaluate the immunological and toxicity response associated with infection with the engineered HMGA-6 *hyper binding site*.

### Monitoring of mouse body weight and other behavioral changes

**Fig 1:**
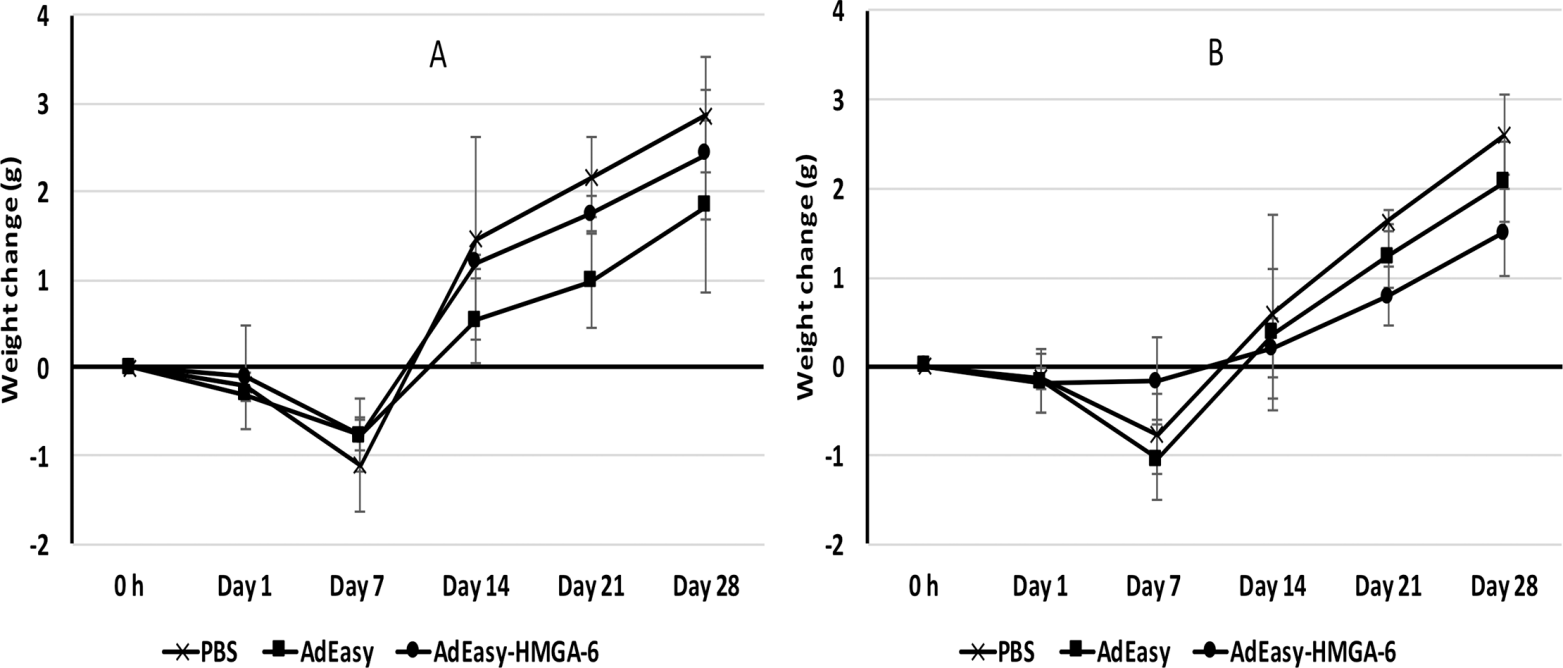
Change of body weight after direct injection of virus into the pancreas or liver. (A) Change of mice body weight after injecting the AdEasy or AdEasy-HMGA-6 virus into the liver. (B) Change of mice body weight after injecting the AdEasy or AdEasy-HMGA-6 virus into the pancreas. Mice were injected with 1.0X10^8^ virus particles/kg of body weight. Data are shown for the 30-day time-period group. Mouse body weight was measured before injection of the viruses or 20 μL PBS (as vehicle control) and monitored until euthanization at indicated time points. The body weight change (on vertical axis) indicates the difference between the weight before injection and the weight at indicated time points. All data represent the means ± SD of three mice.

Mouse body weight was monitored for changes after surgery and compared to the control group weight. **Fig 1** shows that the body weight started to decrease in case of all treatment groups (even when PBS was injected) and the maximum weight loss was observed up to 7 days post-injection. By the day 14, body weights started to increase gradually and the mice continued to gain weight until they were sacrificed. The transient loss of the body weight could be attributed to the surgical trauma that the mice experienced during intra-organ injection. However, the mice injected in the pancreas or liver did not show any behavioral changes that indicate stress or pain.

### Biodistribution of adenovirus following injection into liver or pancreas

**Fig 2:**
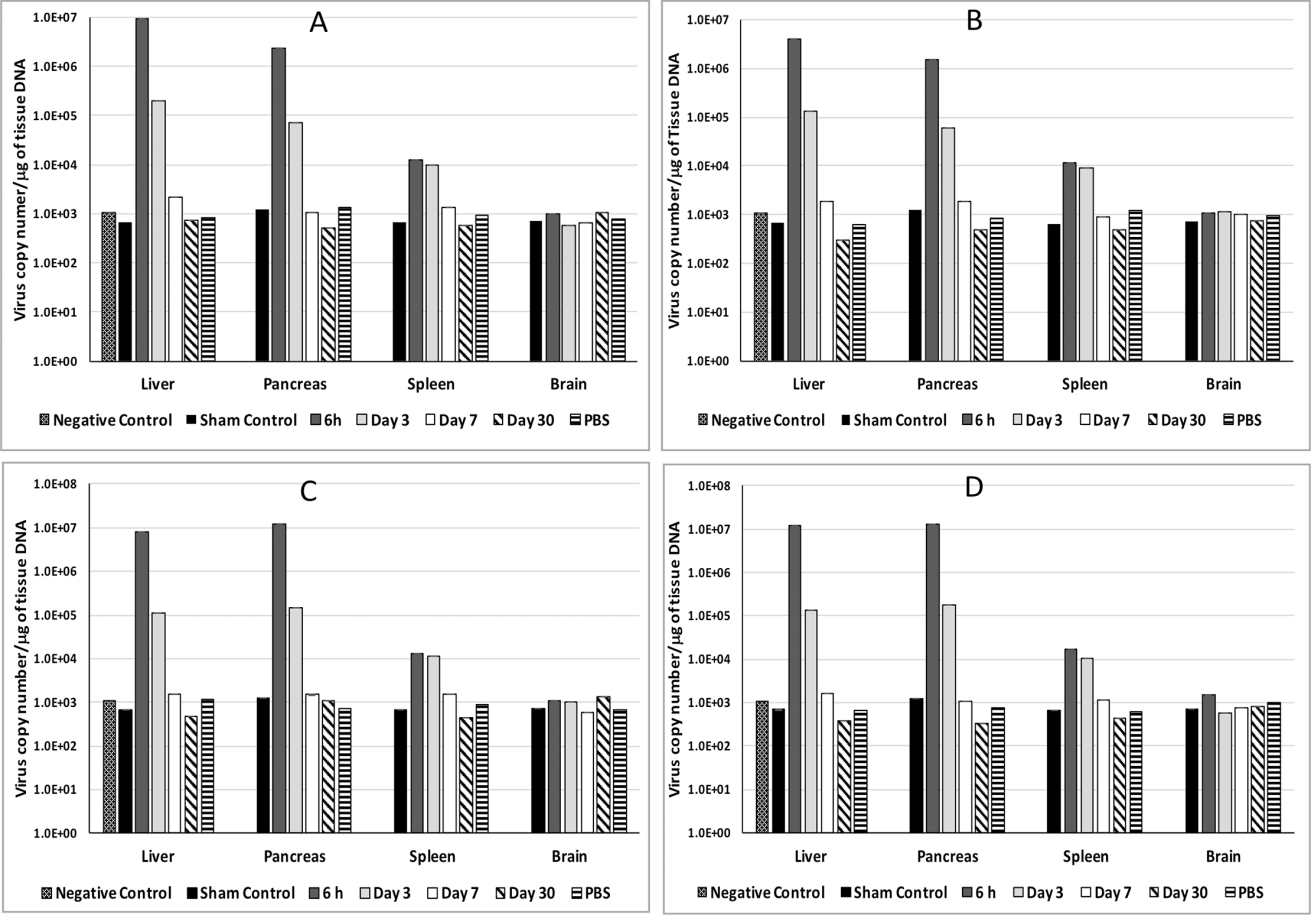
Determination of virus copy number after injecting virus into the pancreas or liver. (A) Viral copy number in different organs after injecting AdEasy into the liver. (B) Viral copy number in different organs after injecting AdEasy-HMGA-6 into the liver. (C) Viral copy number in different organs after injecting AdEasy into the pancreas. (D) Viral copy number in different organs after injecting AdEasy-HMGA-6 into the pancreas. Mice were injected with 1.0X10^8^ virus particles/kg of body weight. Viral copy numbers were determined by qRT PCR using five microliters of total genomic DNA isolated from each tissue as template. The AdEasy and AdEasy-HMGA-6 groups had n = 12 and liver, pancreas, spleen and brain were collected 6h, 3, 7, 10 and 30 days post-injection. Tissues from the vehicle control group (n = 3) were collected after 3 days. The sham control group (n = 3) did not undergo any surgical procedure. Quantification cycle (Cq) values less than the negative control were considered negative. All data represent the means ± SD of three mice.

In order to evaluate the efficiency of the direct injection of virus into liver or pancreas as a potential route of virus administration to treat pancreatic or liver tumors, AdEasy or AdEasy-HMGA-6 virus was injected directly into liver or pancreas and viral DNA was quantified by qRT PCR as a measure of the biodistribution of the virus in different organs. We have studied the presence of virus in liver, pancreas, spleen, and brain following injection. Virus was detected in all tested organs except brain (**Fig 2**). There was no significant difference in the distribution pattern between the AdEasy or AdEasy-HMGA-6 virus (**Fig 2**). In case of both viruses, the highest viral copy number was detected in liver when the viruses were injected directly into the liver (**Fig 2A** and **2B**). On the other hand, when viruses were injected into pancreas, there was no significant difference in terms of viral distribution between liver and pancreas (**Fig 2C** and **2D**). In all cases, lowest concentration of virus was detected in spleen. In addition to the viral biodistribution, **Fig 2** shows the rate of clearance of the AdEasy and AdEasy-HMGA-6 virus following injection. Mice were euthanised 6 h, 3 d, 7 d, and 30 d following injection and liver, pancreas, spleen, and brain samples were examined for the presence of viral DNA. qRT-PCR data shows that highest viral copy number was detected 6h post-injection for both AdEasy and Adeasy-HMGA-6 viruses (**Fig 2**). Viral copy number decreased by 10 to 100 times by day 3 and became undetectable by day 7 or 30 in all cases.

### Determination of immune response follwing virus injection

**Fig 3:**
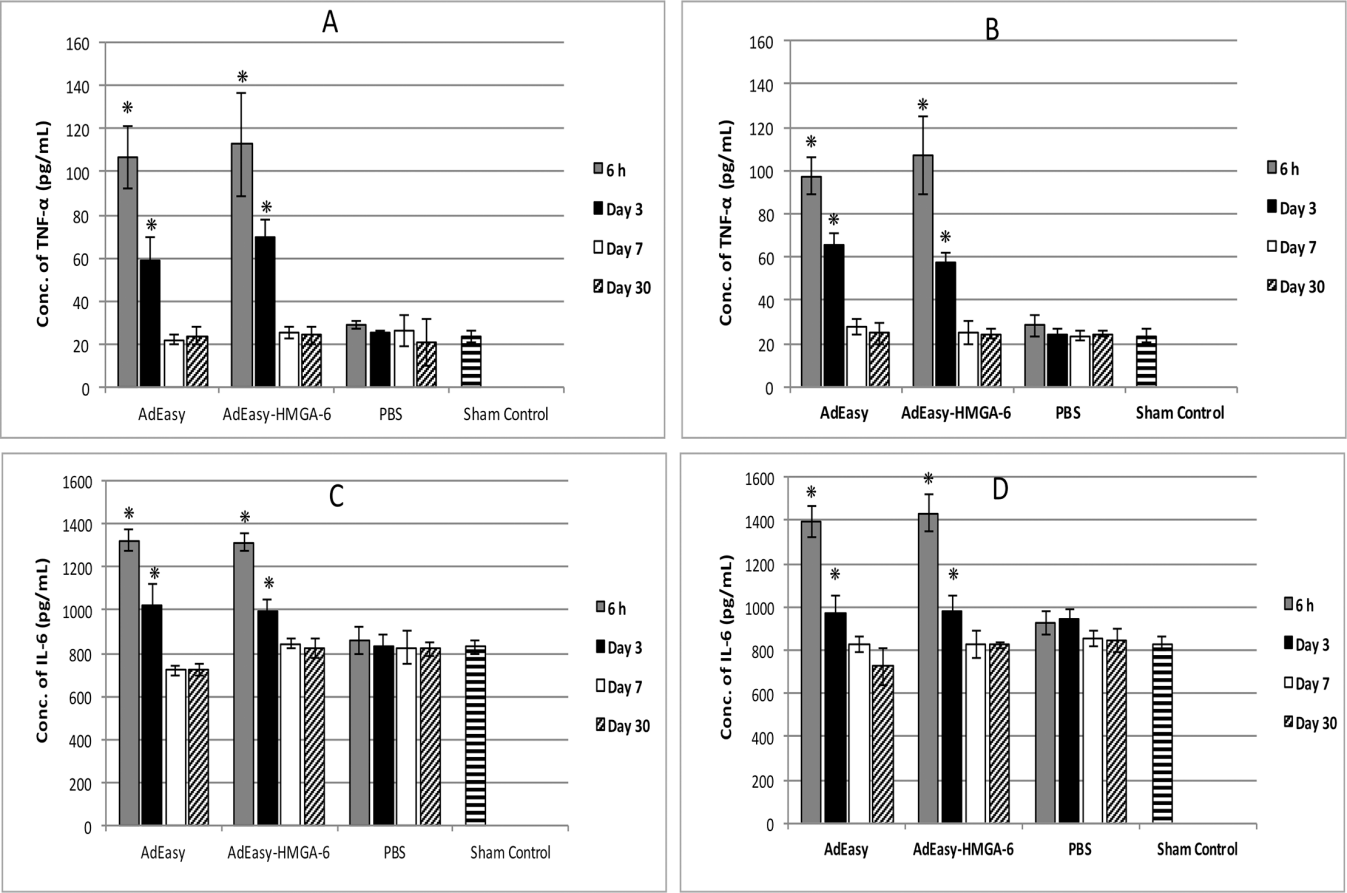
Determination of serum TNFα and IL-6 following injection with virus. (A) Serum levels of TNFα following virus injection into liver. (B) Serum levels of TNFα following virus injection into pancreas. (C) Serum levels of IL-6 following virus injection into liver. (D) Serum levels of IL-6 following virus injection into pancreas. Serum levels of TNFα or IL-6 were determined by ELISA following injection 1.0X10^8^ virus particles / kg of body weight or PBS (20μL) as vehicle control. Serum samples were collected 6h, 3, 7 or 30 days post-injection of viruses or PBS. The sham control group did not undergo any surgical procedure. All data represent the means ± SD of three mice.

In order to examine if the presence of the HMGA-6 *hyper binding site* elicited any additional immune response compared to the native virus, serum samples were analyzed to quantify TNFα and IL-6 levels following injection of the AdEasy or AdEasy-HMGA-6 virus into liver or pancreas. In the case of either liver or pancreas injection, both viruses caused significant (*p* < 0.05 in all cases) increase of serum TNFα and IL-6 levels for up to 3 days (**Fig 3**). The highest levels of TNFα and IL-6 were observed 6 h post-injection and the concentrations returned to normal by day 7 or day 30 when compared to the sham or vehicle control group. Following injection of both viruses (either into liver or pancreas), the TNFα concentration increased up to ~5 times and ~3 times within 6h and 3 days of injection, respectively in comparison to the sham control group (*p* < 0.05 in all cases) (**Fig 3A** and **3B**). Notably, there was no significant difference in the serum TNFα levels between the AdEasy and AdEasy-HMGA-6 virus when analysed 6h of post-injection into liver (*p* = 0.392) or pancreas (*p* = 0.271). Similar trends were observed after 3 days of injection into liver (*p* = 0.166) or pancreas (*p* = 0.108). The serum IL-6 concentraion increased up to 65% and 20% in case of 6h and 3 days of post-injection respectively and this increase was significantly higher than the sham (*p* < 0.05 in all cases) or vehicle control (*p* < 0.05 in all cases) group (**Fig 3C** and **3D**). Similar to the TNFα concentration, the introduction of the HMGA-6 *hyper binding site* did not cause additional increase of IL-6 levels. As shown in **Fig 3C** and **3D**, there was no significant difference in serum IL-6 level between the AdEasy and AdEasy-HMGA-6 virus when analysed 6h of post-injection into liver (*p* = 0.41) or pancreas (*p* = 0.324) or 3 days of post-injection into liver (*p* = 0.353) or pancreas (*p* = 0.488). These data provide evidence that introduction of the HMGA-6 *hyper binding site* into the AdEasy vector did not cause any additional increase of immune response compared to the empty vector (AdEasy) itself.

### Determination of liver toxicity following virus infection

**Fig 4:**
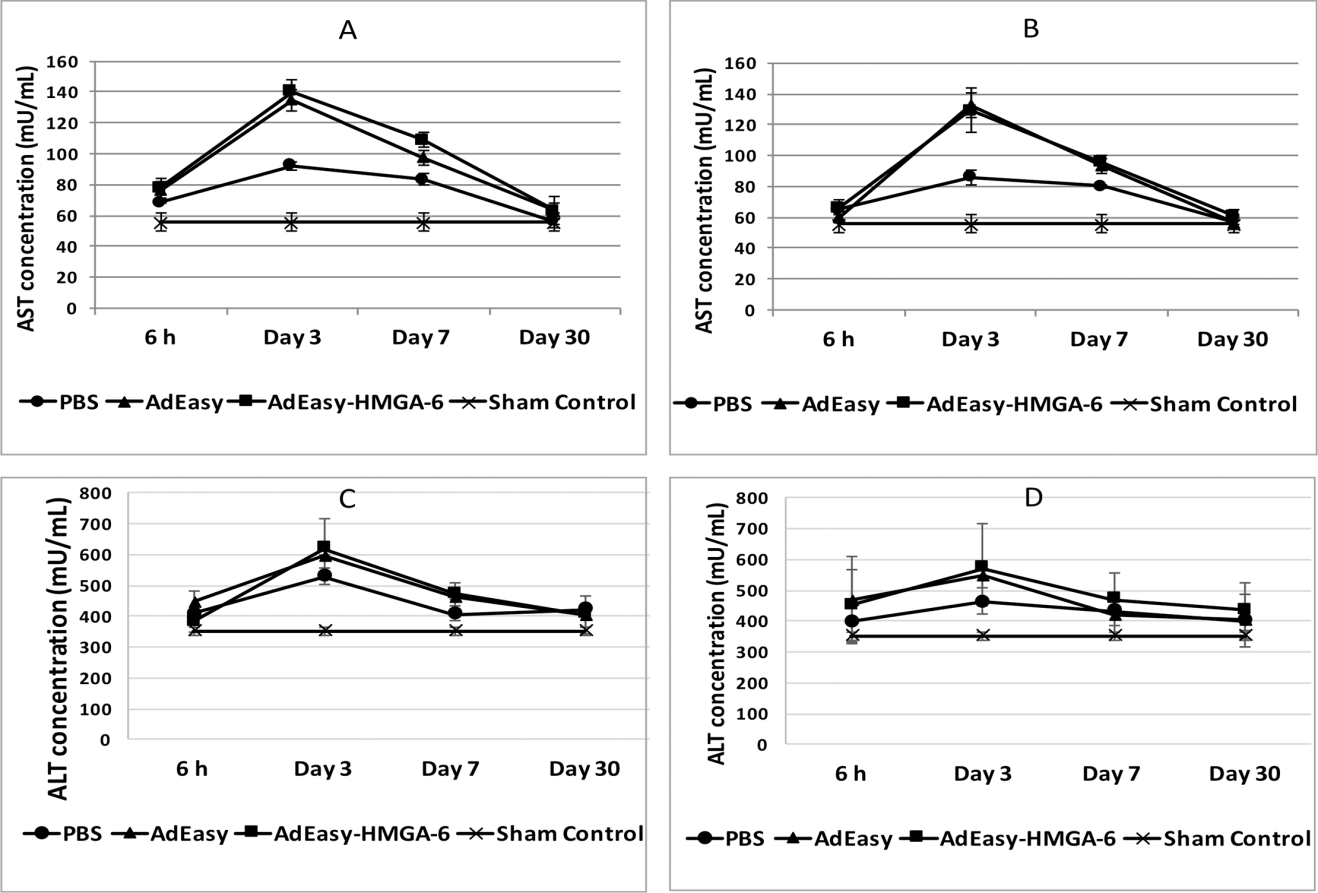
Analysis of serum transaminases following injection with viruses. (A) ALT concentration following injection with AdEasy, AdEasy-HMGA-6 or PBS as vehicle control into the liver. (B) ALT concentration following injection with AdEasy, AdEasy-HMGA-6 or PBS as vehicle control into the pancreas. (C) AST concentration following injection with AdEasy, AdEasy-HMGA-6 or PBS as vehicle control into the liver. (D) AST concentration following injection with AdEasy, AdEasy-HMGA-6 or PBS as vehicle control into the pancreas. Viruses were injected with a dose of 1.0X10^8^ virus particles / kg of body weight. 20μL of PBS was injected as a vehicle control. The sham control group did not undergo any surgical procedure. Serum samples were collected 6h, 3, 7 or 30 days post-injection of viruses or PBS. All data represent the means ± SD of three mice.

The role of the HMGA-6 *hyper binding site* in inducing liver toxicity was evaluated by determining the serum ALT and AST levels following injection of the AdEasy or AdEasy-HMGA-6 virus (1.0X10^8^ vp/Kg of body weight) into mice liver or pancreas. Mice were euthanised 6 h, 3 d, 7 d, and 30 d following injection and serum samples were analyzed to determine the ALT and AST concentrations. When the AdEasy or AdEasy-HMGA-6 virus was injected into either liver or pancreas, there was an increase of ALT and AST levels after 6 h of injection and the highest concentrations were observed after 3 days which was followed by a steady decrease to normal level within 30 days of injection (**Fig 4**). There was no significant difference in the pattern of this transient liver toxicity between the AdEasy or AdEasy-HMGA-6 virus when injected into either of the organ (*p* > 0.05 in all cases for both ALT and AST). Notably, injection of the vehicle (PBS) into liver or pancreas also caused some liver toxicity as indicated by significantly higher ALT (*p* = 0.001 for both liver and pancreas injection) and AST (*p* = 0.002 for both liver and pancreas injection) levels.

## Discussion

In our previous study, we have engineered a replication defective Ad containing a synthetic DNA elements (AdEasy-HMGA-6) that effectively binds to HMGA protein and caused reduced cancer cell growth and increased chemotherapy susceptibility [27]. Although, low or undetectable HMGA expression has been reported in normal adult mice [21], it is possible that inhibition of HMGA function by our engineered decoy HMGA-6 *hyper binding site* could alter gene regulation or cause other complications in the host organism. Therefore, in this study, we conducted initial preclinical experiments to determine if delivery of the decoy HMGA-6 *hyper binding site* via the Ad vector (AdEasy-HMGA-6) caused any unanticipated toxic effects compared to the empty Ad vector (AdEasy) in female C57BL/6 mice. We also studied the biodistribution of our engineered vector following injection into liver or pancreas. Previous studies with recombinant Ad for cancer treatment showed that systemic administration of the Ad by intravenous injection is linked to increased toxicity in liver and other organs compared to intratumoral injection [31, 32]. Furthermore, systemic virus delivery may result in inefficient infection of cancer cells due to the poor interstitial penetration in solid tumors [31, 33]. Therefore, we have examined direct injection of the recombinant virus into liver or pancreas as an alternative mode of delivery that is intended to mimic direct injection of the virus into a tumor in either the pancreas or liver.

We detected the presence of the recombinant viruses for up to 3 days of post-injection and complete removal by 7 days in case of both liver and pancreatic injection. Following injection into liver or pancreas viral DNA was detected in liver, spleen and pancreas which suggests that virus could be distributed to other organs via the circulatory system. We did not detect viral DNA in brain for either injection method which was predictable since the blood brain barrier restricts systemic distribution of Ad into brain [34, 35]. There was no difference in terms of viral distribution between AdEasy and AdEasy-HMGA-6 viruses which suggested that insertion of the HMGA-6 *hyper binding site* into the AdEasy vector did not impact on the viral dissemination.

The major safety issue associated with the Ad vector is the activation of both the innate and adaptive immune response by the vector [36]. Activated cytokines, especially TNFα and IL-6 play a crutial role in activating innate immune response against Ad infection [37]. Systemic administration of Ad vector has been shown to activate the nuclear factor NFκB in liver within 15 minutes of virus administration and stimulate TNFα and IL-6 release as early as 3 h [38]. Elevated level of TNFα in response to viral administration has been found to be associated with the inhibition of viral replication and stimulation of apoptosis of virally infected cells [37]. In addition, TNFα stimulates IL-6 transcription in response to viral administration in hepatocytes, kupffer cells, and other cell types [39, 40]. Moreover, IL-6 activates cytotoxic T lymphocytes (CTL) which is responsible for the clearance of virally infected cells [41]. Therefore, to evaluate the immune response associated with our synthetic HMGA-6 *hyper binding site*, serum TNFα and IL-6 levels were compared between AdEasy-HMGA-6 or AdEasy injected mice. Our results showed that injection of both viruses caused significant increase of serum cytokines level for up to 3 days of post-injection and then decreased to normal levels within 7 days which is consistent with some other previous studies [42, 43]. However, the important finding of this study is that the addition of the decoy HMGA-6 *hyper binding site* did not cause any additional toxicity compared to the empty vector. This result was not unexpected because several previous studies showed that majority of the Ad vector related toxicity was attributed to the immune response against the vector encoded foreign protein [44, 45]. Since our synthetic HMGA-6 *hyper-binding site* does not encode any foreign protein, the AdEasy-HMGA-6 virus did not elicit significantly different immune response compared to the AdEasy virus.

Numerous studies have supported that administration of Ad vector in mice is associated with the increased level of cytokines and chemokines, especially TNFα and IL-6 which in turn cause inflammatory response in liver as well as recruitment of immune effector cells such as neutrophils, macrophages and natural killer (NK) cells, resulting in acute liver damage [43, 46, 47]. In response to liver damage, elevated levels ALT and AST have been reported in several previous studies [28, 47, 48]. Consistent with these studies, our results also suggested that administration of AdEasy or AdEasy-HMGA-6 into liver or pancreas was associated with higher inflammatory cytokines levels as well as serum transaminases levels. Our data showed that the highest levels of TNFα and IL-6 were detected 6 h of post-administration and the highest transaminases levels were measured 3 days post-injection and the highest copy number of viral DNA was detected in liver. These observations indicate that Ad induced elevation of TNFα and IL-6 level which then causes inflammation in liver, resulting in increased serum transaminase levels. This finding is consistent with the study of Koizumi *et al*., which showed that inhibition of IL-6 signalling pathway following Ad aministration resulted in reduced liver toxicity in mice [47]. We also observed that engineering of the HMGA-6 *hyper binding site* in the AdEasy vector did not induce additional liver damage as indicated by the comparable level of serum transaminases in case of both viruses injection.

The results of the current study aid in understanding viral distribution post-injection into target organs and also provide greater insight into the complex Ad-mediated immune response *in vivo*. In addition, the study indicated that there is no increased immune response to the presence of the engineered decoy HMGA-6 *hyper binding site*. Engineering a virus capable of sequestering oncogenic transcription factors in cancer cells is a promising and selective treatment option that has the potential to be utilized for a variety of cancers. If the engineered AdEasy HMGA-6 virus is able to safely deliver the decoy HMGA-6 *hyper binding site* to the nucleus of cancer cells without initiating a life threatening immune response, this technique may provide an effective and novel clinical treatment for cancer therapy.
